# Case of Complete Section of the Trachea and Œsophagus

**Published:** 1830-07-01

**Authors:** 


### XXXIV.

CASE OF COMPLETE SECTION OF THE TRACHEA AND ŒSOPHAGUS, WITH RECOVERY, EXCEPTING AN AERIAL FISTULA. By Dr. LUDERS.—(Journal Complement. April, 1830.)

A man, 37 years of age, of athletic constitution, being pursued by the peace-officers, cut his throat (with a pruning knife) in a very extensive manner, and was found lying on his face weltering in blood. He was immediately conveyed to the hospital. The trachea was found to be severed across, between the first and second cartilages, as also the œsophagus, the wound penetrating to the bodies of the cervical vertebræ, without wounding any of the great vessels or nerves. The muscles only suffered, and even the sterno-cleido muscles escaped. The wretched suicide had plunged the crooked point of the knife into the neck at one side of the trachea, then pushed it under that tube and the œsophagus, and, by drawing the instrument outwards, severed the alimentary and aerial conduits completely! The patient was pallid, deprived of speech, and could breathe only when lying forward on the belly, so as to permit the blood to flow out by the external wound. The man violently rejected all assistance, and passed the night in the position above mentioned. Dr. Luders having ascertained the total section of the trachea and œsophagus, prognosticated the death of the man by starvation. Nevertheless, he endeavoured to introduce an elastic canula into the œsophagus, for the purpose of conveying some milk into the stomach; but was prevented by violent efforts to vomit, and by threatenings of suffocation. As he complained much of thirst, any liquid taken into the mouth passed out by the wound, or trickled into the trachea and excited vomiting.

*Second day.* Dr. L. attempted to re-unite the divided extremities of the trachea by means of ligatures; but was entirely foiled by the convulsive efforts to cough. The patient had expectorated a large quantity of bloody mucus in the night, and was this day able to lie on his back, making signs of hunger. Some gruel and laudanum were thrown up per anum, and an instrument was ordered to be made for conveying nutriment into the stomach through the œsophagus.

*Third day.* The man had slept some hours between the paroxysms of coughing. He was free from fever; and, setting aside the hunger, he appeared to be comfortable. The point of the instrument being introduced into the œsophagus, some milk and egg was passed into the stomach, by which the sense of hunger was a little diminished. Another attempt at re-uniting the divided trachea failed, as might be expected.

*Fourth day.* The patient had slept well, and felt himself better to-day. On taking some milk into his mouth, a little of it escaped down into the stomach, while bending his head forwards on the chest. On the fifth day, the power of swallowing was still farther increased—the exterior wound was diminishing, and the patient took a cup of flour and milk boiled, the greater part of which went down into the stomach. We need not pursue the details in the minute manner which the writer has adopted. It is sufficient to state that the power of swallowing is restored—and that the patient breathes through a tube in the trachea, which has now been continued there for the space of two years. This forms the third or fourth instance of the same fistulous communication, rendered necessary for the preservation of life. We put the first on record 15 years ago, and the patient (Mr. Price, of Portsmouth) still breathes through the tube.

Ambrose Paré mentions the case of a man, whose trachea and œsophagus were totally severed by the stroke of a poniard, and who died on the fourth day from the accident. The great vessels escaped. Placentius alludes to a case, where the trachea and œsophagus were both wounded by the weapon of a suicide, the jugulars and carotids remaining secure. The patient recovered in a month. Other instances are related in the writings of Helwigg, Purmaun, Starck, Debruck, Kurtzwigg, &c. We have seen a case where the trachea was totally, and the œsophagus partially, severed, in the person of a Malay, and where recovery was complete in six or seven weeks. The case is detailed in an early number of this Journal. Such instances may guard our prognoses against too much despondency, and even assist our modes of treatment, under similar circumstances.

---

